# Characterization and immunoprotection of thioredoxin reductase TrxB knockout mutant of *Salmonella* Enteritidis

**DOI:** 10.3389/fcimb.2025.1659729

**Published:** 2025-09-17

**Authors:** Siping Zhu, Lili Wang, Hong Li, Chihuan Li, Xintong Zhu, Chao Ren, Xiaochen Liu, Yulai Dong, Qiumei Shi, Zhiqiang Zhang

**Affiliations:** ^1^ Hebei key laboratory of preventive veterinary medicine, Hebei Normal University of Science & Technology, Qinhuangdao, China; ^2^ Weichang Man and Mongolian Autonomous County Xinrui Agricultural Development Ltd., Chengde, China

**Keywords:** *Salmonella* Enteritidis, TrxB, gene deletion, virulence, vaccine

## Abstract

**Background:**

*Salmonella* Enteritidis (*S*. Enteritidis) is an important zoonotic pathogen that poses a major threat to animals and human health. TrxB, as a key component of the thioredoxin system, is a thioredoxin reductase ubiquitously present in organisms. It is mainly involved in maintaining cellular redox balance, but its role in the pathogenicity of *S*. Enteritidis remains unclear.

**Methods:**

In this study, we generated a *trxB*-deficient strain from *S*. Enteritidis C50336 strain to investigate how TrxB affects the biological characteristics and pathogenesis of the bacterium. The virulence of Δ*trxB* was assessed by measuring Δ*trxB* resistance to environmental stress, biofilm formation ability, motility, adhesion, invasion ability, intracellular survival, LD_50_, virulence gene expression levels, and *in vivo* colonization ability. Additionally, the study measured specific IgG antibody levels in mice, lymphocyte proliferation, and the immunoprotective effect of Δ*trxB*.

**Results:**

We found that deletion of *trxB* gene did not affect the growth and biochemical properties of the *S*. Enteritidis strain but significantly reduced its motility, drug resistance, biofilm formation, and tolerance to environmental stress. After *trxB* knocked out, the adhesion and invasion capacities of *S*. Enteritidis to Caco-2 cells, along with its proliferation in RAW264.7 cells, were significantly reduced. Additionally, the *trxB*-deficient strain exhibited significantly lower pathogenicity than the parental strain, evidenced by a more than 100-fold increase in LD_50_. We also observed a significant decrease in the expression of virulence-related genes in the *trxB*-knockout mutant. More importantly, immunization with this deletion strain can confer promising protection against challenge with the C50336 strain.

**Conclusion:**

These findings indicate that TrxB is a crucial virulence factor in *S*. Enteritidis, playing critical roles in its pathogenicity.

## 1 Introduction

*S*. Enteritidis, a common zoonotic pathogen, can cause gastroenteritis and systemic infections in humans and various animals. These infections result in high morbidity and harm, posing a significant threat to livestock production and human health (Knodler and Elfenbein, 2019). *S*. Enteritidis is mainly transmitted via the fecal-oral route. During infection, various environmental stresses and host immune responses may affect its infectivity. Over time, *S*. Enteritidis has evolved to evade host defenses by expressing various virulence factors that aid its adaptation and replication within the host (Coburn et al., 2007; Quan et al., 2019; Takaya et al., 2019).

The thioredoxin system (Trx) is a widespread antioxidant enzyme system found in all living organisms. It plays a crucial role in maintaining cellular redox balance and facilitating redox-mediated signal transduction (Kanzok et al., 2001; Branco et al., 2014; Lu and Holmgren, 2014a; Ren et al., 2018). Additionally, the Trx system was also reported to be a thiol- and protein-repair system, protecting organisms from protein inactivation caused by cysteine residue oxidation under oxidative conditions (Kreimer et al., 1997). In tumor research, TrxB has been demonstrated to be an effective cancer therapy target due to its critical role in maintaining cellular redox homeostasis and redox-mediated signal transduction. In bacteria, TrxB has been reported to be involved in removing reactive oxygen species in cells, thereby benefiting bacterial survival under oxidative stress conditions (Kuhns et al., 2015; Chupakhin and Krasavin, 2021). As a typical intracellular pathogen, *Salmonella* encounters various stressors, including reactive oxygen species (ROS) and reactive nitrogen species (RNS), in macrophages and epithelial cells during infection (Kobayashi, 2019). In a recent study, oxidative stress was also reported to stimulate the transcription of *Salmonella* pathogenicity island-2 (*SPI*-2) genes, thereby affecting its replication within macrophages (Kim et al., 2022). Therefore, we hypothesized that TrxB plays a significant role in the pathogenicity of *S*. Enteritidis. In the present study, we generated a Trx-inactivated strain of *S*. Enteritidis by knocking out the core enzyme TrxB and assessed the effect of this protein on the pathogenicity of *S*. Enteritidis through comparative analysis of virulence-related biological properties.

## 2 Materials and methods

### 2.1 Bacterial strains, cells and plasmids

*S*. Enteritidis C50336 was isolated from the feces of a patient with diarrhea and purchased from the National Institute for the Control of Pharmaceutical and Biological Products (China). It was kept in the Key Laboratory of Preventive Veterinary Medicine, Hebei Province. Unless otherwise stated, the strains used in this study were cultured in Luria broth (LB) liquid medium(Haibo Biotechnology Co., Ltd., HB0128, China) (37 °C, 180 r/min). The cells used in this study, Caco-2BBE cells and RAW264.7 cells, were both maintained in the Key Laboratory of Preventive Veterinary Medicine of Hebei Province. They were cultured in a CO_2_ incubator with 5% CO_2_ using high-sucrose Dulbecco’s Modified Eagle Medium (DMEM) (Thermo Fisher Scientific Co., Ltd., USA) supplemented with 10% fetal bovine serum (Thermo Fisher Scientific Co., Ltd., China) and 1% Penicillin-Streptomycin Solution (Beijing Solarbio Science & Technology Co., Ltd., China). Gentamicin (100 μg/mL) was added to the medium when necessary. The plasmids pKD3, pKD46, pBR322 and pCP20 for bacterial gene knockout were provided by Invitrogen.

### 2.2 Experimental animals

Kunming mice (6–8 weeks old) were purchased from Beijing Speifu Biotechnology Co., Ltd. (Beijing, China) and kept according to standard protocols for animal experiments.

### 2.3 Construction of a *trxB* gene deletion strain and a complementary strain of *S. Enteritidis*

The *trxB* gene-deleted strain was constructed using the λ-Red homologous recombination technique. The plasmid pKD3 served as a template to amplify the *cat* targeting gene fragment using P1/P2 primers (**Table 1**). This fragment was then electrotransformed into C50336-pKD46 receptor cells. The strain was identified by PCR using P3/P4 primers (**Table 1**). Subsequently, the strain was incubated in a water bath at 42°C for 5 to 6 hours to eliminate pKD46. Finally, the deletion strain C50336Δ*trxB*::*cat* containing the *cat* gene was obtained. The extracted genome of the C50336 strain was used as a template to amplify the *trxB* gene fragment by PCR using P5/P6 primers (**Table 1**). This fragment was cloned into the pBR322 plasmid to construct the recombinant plasmid pBR322-*trxB*. After sequencing confirmed the correct plasmid, it was electrotransformed into the Δ*trxB* strain. The complementary strain, named Δ*trxB*+*trxB*, was then constructed and verified by PCR and sequencing using P3/P4 primers. The purified PCR product was sent to Sangon Biotech (Shanghai) Co., Ltd. (China) for sequencing. The *trxB* gene expression in Δ*trxB* and Δ*trxB*+*trxB* was confirmed by qPCR. Briefly, RNA of each strain was extracted using a bacterial RNA extraction kit (Beijing Aidlab Biotechnologies Co., Ltd., RN63, China), reverse transcribed into cDNA, and subjected to qPCR verification using primers P7 and P8 (**Table 1**) to assess the expression of the *trxB* gene.

**Table 1:** Primers used for constructing the mutant and the complemented strain.

Primer	Sequence (5’-3’)
P1	CTCATGGGCACGACCAAACACAGTAAGCTGCTTATTCTGGGCTCAGGACCGTGTAGGCTGGAGCTGCTTCG
P2	AAAGTCCGGCGATGTCCAGCGTCTCGATATTATCGCTCTGCTGCGTGTCGATGGGAATTAGCCATGGTCC
P3	TATGTCCGCAGTTACGTTGGC
P4	CAGGATACCTCTTACACCCTCACC
P5	CGGGATCC ATGGGCACGACCAAACACAGTAAGC
P6	GCGTCGACCTATTTGCTCGCGTCGGCCAGA
P7	CGATAGACCGAAACCCAGAG
P8	GCGGAGGACAAAGACCAGTA

### 2.4 Growth curve analysis

The strains were cultured overnight, and then transferred into LB and M9 medium with a 1:50 dilution on the next day, and cultured at 37°C with shaking at 180 rpm. M9 medium consists of 17 g Na_2_HPO_4_·7H_2_O, 3 g KH_2_PO_4_, 0.5 g NaCl and 1.0 g NH_4_Cl. The OD_600_ was measured every hour for 10 hours by sampling the culture. Finally, the growth curve was plotted to analyze the strains’s growth characteristics.

### 2.5 Bacterial motility assay

Bacterial motility was analyzed on semi-solid plates following the method described in literature (Duan et al., 2012). Five microliters of overnight culture was punctured into the center of the semi-solid medium plate and incubated at 37 °C for 5 h. The bacterial motility was assessed by measuring the diameter of growth on the semi-solid medium plate.

### 2.6 Detection of biofilm formation

Referring to the literature (Dong et al., 2011), bacterial biofilms were measured using crystal violet staining for both observation and quantitative analysis. Each strain was inoculated into 5 mL of liquid medium and incubated at 30 °C for 3 days. The bacterial cultures were removed, and the biofilms attached to the tubes were washed three times with PBS. Then, they were stained with 2% crystal violet solution for 15 minutes. The crystal violet bound to the biofilm was dissolved in anhydrous methanol to determine OD_570_ nm values for quantifying biofilm formation. This experiment was repeated three times independently.

Each strain was inoculated onto medium containing Congo red (160 mg/L) and Caulmers Brilliant Blue (10 mg/L), then incubated at 28°C for 48 hours. The morphology and color of the colonies of each strain were observed. Additionally, each strain was inoculated onto medium containing 200 mg/L fluorescent whitening agent and incubated at 28°C for 48 hours. The fluorescence intensity of the colonies was compared under D366 nm ultraviolet light to that of the fluorescent whitening agent alone, in order to analyze cellulose production in the biofilm.

### 2.7 Determination of minimum inhibitory concentration of various antimicrobials

The minimum inhibitory concentration was determined following the Clinical and Laboratory Standards Institute guidelines (Clinical and Laboratory Standards Institute, 2009). The studies on the relevance of Trx system and antibiotic resistance are very limited, thus the choice of antibiotics in this study was random. In this study, 12 antimicrobials commonly used in clinical practice, including penicillin, acetylmethquine, cefotaxime, cefuroxime, ampicillin, cotrimoxazole, norfloxacin, ciprofloxacin, enrofloxacin, ofloxacin, lincomycin, and fosfomycin (Shanghai Yuanye Bio-technology Co., Ltd., China), were selected to perform the MIC test. Each overnight culture was adjusted to the same concentration (1×10^6^ CFU/mL). Each antimicrobial was serially two-fold diluted in test tubes containing sterile Mueller Hinton (MH) medium. Then, equal amounts of the adjusted bacterial solution were added to the serially diluted antimicrobials. The mixtures were incubated at 37 °C for 16–18 hours. *E. coli* ATCC25922 was used as the control strain. The experiment was repeated three times.

### 2.8 Stress tolerance assay

Bacterial stress tolerance was assessed by measuring the survival of each strain under stress conditions as previously described (Bringer et al., 2005). Briefly, bacteria in the logarithmic growth phase were exposed to different stresses, including acidic stress (pH 3.5), alkaline stress (pH 10.5), heat stress at 42 °C, hypertonic stress (2.5 mol/L NaCl), and hypotonic stress induced by deionized water for 1 hour, as well as oxidative stress (10 mmol/L H_2_O_2_) for 10 minutes. After treatment, colonies were counted using serial dilution, and the survival rate of each strain was calculated as the ratio of colony-forming units (CFU) after stress exposure to the CFU before treatment. Three independent replicate experiments were used.

### 2.9 Adhesion, invasion and intracellular survival assays

The Caco-2BBE cells line, derived from colon adenocarcinoma, was used to assess bacterial adhesion and invasion abilities (Kortman et al., 2012). Caco-2 cells were seeded in 6-well plates, and a bacterial suspension in the logarithmic growth phase was added to the wells at a multiplicity of infection (MOI) of 100. The cells were centrifuged at 1000 rpm for 5 min and then incubated at 37 °C for 1 h. Cells were washed three times with PBS, lysed with 1% Triton X-100, and the lysates were collected for bacteria counting. Adhesion rate = (number of adhered bacteria/number of bacteria in the inoculum per well) × 100%.

For the invasion assay, the infection method was the same as that of the adhesion assay. After incubation for 1 h at 37 °C in a 5% CO_2_ incubator, the medium was replaced with DMEM containing gentamicin (100 μg/mL), and incubation continued for another hour. Then, cells were lysed by adding 1 mL of 1% Triton X-100. Invasion rate = (number of intracellular bacteria/number of bacteria in the inoculum per well) × 100%.

The bacterial intracellular survival in macrophage was determined using cell line RAW264.7, a mouse macrophage cell line. Similar to the adhesion and invasion assays, RAW264.7 cells were infected with bacteria at a MOI of 100 and cells were incubated for 1h and then the medium was switched to DMEM containing gentamicin (100 μg/mL) to continue incubation. The cells were lysed at 3 and 23 hours post-infection (hpi), and the lysates were collected for bacterial enumeration. Bacterial survival and proliferation within macrophages were determined by calculating the ratio of bacterial counts at 23 hpi to those at 3 hpi.

### 2.10 Bacterial virulence evaluation

Fifty-five Kunming mice were randomly divided into 11 groups, with 5 mice in each group. Five groups were i.p. challenged with C50336 at concentration ranging from 2.1×10^8^ to 2.1×10^4^ CFU in 0.2 mL PBS per mouse. Another five groups were i.p. challenged with Δ*trxB* strains at concentration ranging from 1.3×10^10^ to 1.3×10^6^ CFU. The remaining group was challenged with equal volumes of sterile PBS as a control. The onsets of disease and deaths were recorded for 14 consecutive days, and the LD_50_ was calculated according to the method of Reed and Mutch (Park et al., 2020).

To determine the bacterial load in the primary target organs of *S*. Enteritidis, livers, spleens, and lungs were collected from infected mice for quantitative bacterial analysis. Twenty mice were randomly divided into 2 groups with 10 mice in each, and the mice from the 2 groups were inoculated with *S*. Enteritidis wildtype strain and *trxB*-knockout mutant at the dose of 10^6^ CFU per mouse respectively. At 6 h and 48 h post infection, Liver, spleen, and lung tissues were collected under sterile conditions, weighed and homogenized with 1 ml of sterile PBS. The homogenized solution was diluted in a gradient and spread on LB plates for bacterial counting.

### 2.11 Transcriptome analysis

The C50336 and Δ*trxB* strains were scraped from the LB plate into LB liquid medium and incubated with shaking until the OD_600nm_ reached 1-2. Then, the bacteria were washed three times with sterile PBS. The bacterial precipitate was collected and immediately transferred to a liquid nitrogen tank for quick-freezing, followed by storage at -80 °C in an ultra-low-temperature refrigerator. Finally, they were sent to Shanghai Meiji Biological Company. RNA sequencing and bioinformatic analysis was performed using a cloud platform (Shanghai Majorbio Bio-Pharm Technology Co., Ltd., China) based on the data generated by the Illumina platform. The differentially expressed genes (DEGs) were identified as those showing a fold change (Δ*trxB* vs. C50336) greater than 2 or less than 0.5, with a corrected p-value less than 0.05.

### 2.12 RNA extraction and quantitative real-time PCR

RNA was extracted using the Bacterial RNA Extraction Kit (Beijing Aide Biotechnology Co., Ltd., China). gDNA was eliminated during extraction using DNase I from the same company, and reverse transcription was performed using the Reverse Transcription Kit (Bohang Biotechnology Co., Ltd., China). RT-qPCR was performed using SYBR^®^ PreMix Ex Taq II (TaKaRa Biotechnology (Dalian) Co., Ltd., China). Relative gene expression was determined according to the relative critical threshold (Ct) method using a Stratagene Mx3000P system (Agilent Technologies, CA). Data were normalized using the 2^-ΔΔCt^ method to show the relative fold change of 12 virulence genes compared to C50336. The primers used for qPCR are listed in **Table 2**.

**Table 2:** Primers used for qPCR.

Primers	Nucleotide sequences (5′-3′)
*rpoS-*F	TTTTTCATCGGCCAGGATGT
*rpoS-*R	CGCTGGGCGGTGATTC
*csgD-*F	GCCTCATATTAACGGCGTG
*csgD-*R	AGCGGTAATTTCCTGAGTGC
*bcsA-*R	TGGAAGGGCAGAAAGTGAAT
*bcsA-*F	GCCCAGCTTCAGAATATCCA
*ompR-*F	TGTGCCGGATCTTCTTCCA
*ompR-*R	CTCCATCGACGTCCAGATCTC
*pipB-*F	GCTCCTGTTAATGATTTCGCTAAAG
*pipB-*R	GCTCAGACTTAACTGACACCAAACTAA
*hflK-*F	AGCGCGGCGTTGTGA
*hflK-*R	TCAGACCTGGCTCTACCAGATG
rfbHF	ACGGTCGGTATTTGTCAACTCA
rfbHR	TCGCCAACCGTATTTTGCTAA
Primers	Nucleotide sequences (5′-3′)
*spvB-*F	TGGGTGGGCAACAGCAA
*spvB-*R	GCAGGATGCCGTTACTGTCA
*flgG-*F	GCGCCGGACGATTGC
*flgG-*R	CCGGGCTGGAAAGCATT
*invH-*F	CCCTTCCTCCGTGAGCAAA
*invH-*R	TGGCCAGTTGCTCTTTCTGA
*sodC-*F	CACATGGATCATGAGCGCTTT
*sodC-*R	CTGCGCCGCGTCTGA
*ssaV-F*	GCGCGATACGGACATATTCTG
*ssaV-*R	TGGGCGCCACGTGAA
16SF	CCAGGGCTACACACGTGCTA
16SR	TCTCGCGAGGTCGCTTCT

### 2.13 Measurement of immunoprotective efficacy

Thirty Kunming mice were randomly divided into two groups: an immunized group and a control group. The immunized group received orally of the Δ*trxB* strain, while the control group received the same dose of saline on days 0 and 14. Mice were received orally with Δ*trxB* at a dose of 1.3 × 10^8^ CFU and received a booster immunization with the same dose 14 days later.

Serum-specific IgG assay: Indirect ELISA was employed to assess the level of *S*. Enteritidis-specific antibodies in the serum of immunized mice. The C50336 strain was cultured to logarithmic growth stage, collected for lysis and centrifugation, and the supernatant proteins were encapsulated at 1 µg per well. Blood was collected from the tail tip of three mice randomly taken from each group on days 0, 7, 14, 21 and 28 of immunization for serum isolation. The serum was subjected to an ELISA assay with a serum dilution of 1:400 and HRP labelled secondary antibody at a concentration of 1:10,000, and determined for OD_450nm_ after color development.

Spleen index determination: Three mice were randomly selected and weighed on the 7th, 14th, 21st, and 28th days after immunization, and the spleens were picked up and weighed after euthanasia for calculation of the spleen index. The spleen index was calculated by dividing the spleen weight by the body weight of the mouse as a percentage.

Lymphocyte proliferation assay: Three immunized or non-immunized mice were euthanized 14 days after immunization. The spleens of the immunized mice were aseptically isolated and homogenized. The homogenate was then filtered through a 70 μm cell strainer (Beijing Labgic Technology Co., Ltd.) to obtain spleen cells. Red blood cells were lysed using a red blood cell lysis buffer (Beijing Solarbio Science & Technology Co., Ltd.). The spleen lymphocytes were suspended in RPMI 1,640 Medium(Thermo Fisher Scientific Co., Ltd.) supplemented with 10% fetal bovine serum (FBS), 50 μg/mL penicillin, and 50 μg/mL streptomycin. Cell viability was assessed using the trypan blue exclusion test, and cells were counted using a haemocytometer. After cell counting, lymphocytes were seeded into 96-well tissue culture plate at 5×10^5^ cells each well. The lymphocytes were treated by adding bacterial supernatant antigen (7.5 μg/mL), ConA (2 μg/mL) and PBS of equal volume separately and furthered incubated at 37°Cwith 5% CO_2_ for 72 h. Lymphocyte proliferation was measured using the 3-(4,5-dimethylthiazol-2-yl)-2,5-diphenyltetrazolium bromide (MTT) Cell Proliferation and Cytotoxicity Assay Kit (Shanghai Beyotime Biotechnology Co., Ltd.) according to the instructions. Lymphocyte proliferation capacity was evaluated by the stimulation index (SI), calculated as: SI = (OD_450_ of stimulation group − OD_450_ of medium-only group)/(OD_450_ of unstimulated group − OD_450_ of medium-only group).

Relative protection measurement: Twenty KM mice were randomly divided into two groups, each containing 10 mice. Group A was the immunization group, and Group B was the control group. Mice in Group A were received orally with 1.3 × 10^8^ CFU/mouse of Δ*trxB*, followed by a booster immunization with the same dose at 14 dpi. Mice in Group B received an equal volume of PBS following the same immunization schedule. At 28 days post-immunization, mice of groups A and B were challenged with C50336 strain by i.p. injection at the dose of 2.1×10^8^ CFU. The morbidity and mortality of the mice were recorded for 14 days after challenge. The relative protection rate (RPS) was calculated using the formula: RPS = (the mortality of the control group − the mortality of the immunization group)/the mortality of the control group × 100%.

### 2.14 Ethics statement

All animal experiments were conducted in full compliance with international ethical standards and the Experimental Animal Regulation Ordinances (HPDST 2020-17) as stipulated by the Hebei Provincial Department of Science and Technology. The study protocol was reviewed and approved by the Animal Care and Use Committee of Hebei Normal University of Science and Technology.

### 2.15 Statistical analysis

The significance of the differences between C50336 and Δ*trxB* strains in terms of motility, biofilm formation ability, environmental tolerance, invasiveness, adhesion rate, intracellular survival rate, bacterial load, virulence factor detection, and immune protection ability were determined by GraphPad Prism 9.5.0 software was used, along with one-way analysis of variance (ANOVA) combined with t-tests. Data were expressed as mean ± standard error. Significant differences were denoted with an asterisk (*), where *p < 0.05, **p < 0.01, and ***p < 0.001 are considered to represent statistically significant differences in mean values.

## 3 Results

### 3.1 The *trxB* gene deletion does not affect the growth of *S*. Enteritidis

**Figure 1:**
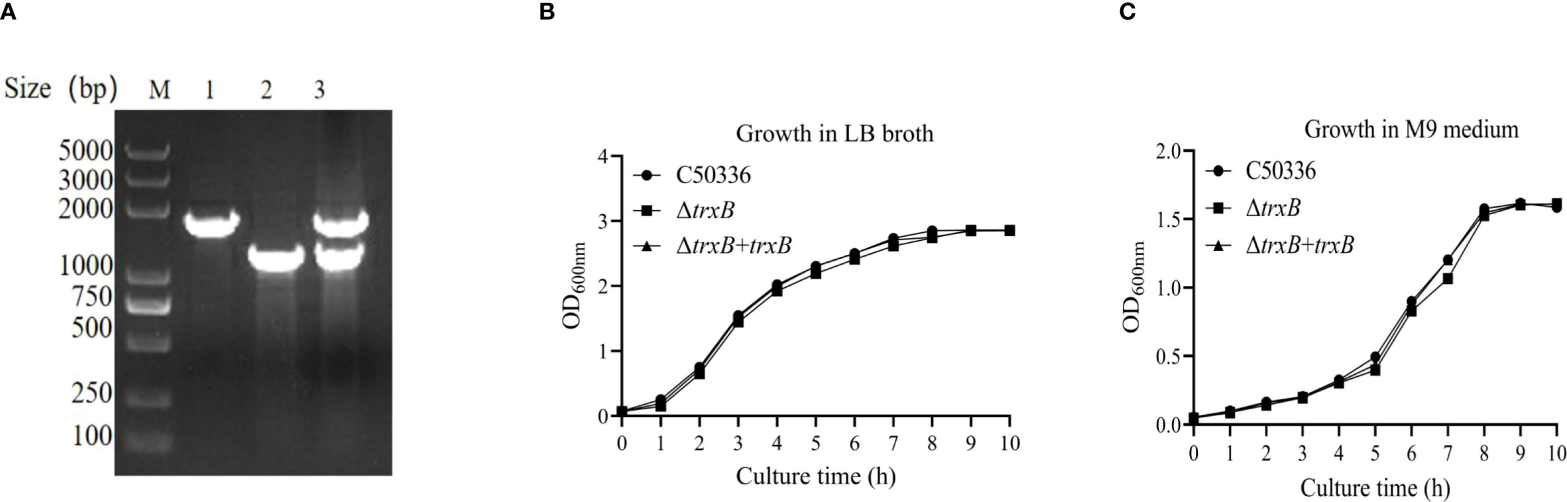
Generation of *trxB* gene deletion strain and its responding complemented strain, and growth curves determination. **(A)** PCR verification of the *trxB* gene deletion strain and Δ*trxB*-complemented strain. The PCR amplification sizes of C50336 and Δ*trxB* were 1764bp and 1234bp respectively. The PCR product of Δ*trxB*+*trxB* showed the both band of C50336 and Δ*trxB* strain. **(B)** Growth curves of C50336, Δ*trxB*, and Δ*trxB*+*trxB* strains in LB broth. **(C)** Growth curves of C50336, Δ*trxB*, and Δ*trxB*+*trxB* strains in M9 medium.

Using λ-Red recombination technology, a *trxB* gene deletion mutant of *S*. Enteritidis C50336 (named Δ*trxB*) and corresponding complemented strain (named Δ*trxB*+*trxB*) were constructed. As shown in **Figure 1A**, the *trxB* knockout mutant and the complemented strain were confirmed by PCR. To access the influence of *trxB* deletion on the growth of *S*. Enteritidis, we examined the growth of each strain in LB medium and nutrient-poor M9 medium. The data showed (**Figures 1B, C**) that all three strains displayed similar growth curves in LB or M9 medium, demonstrating that *trxB* deletion does not affect the growth of *S*. Enteritidis.

### 3.2 The *trxB* gene affects the motility of *S*. Enteritidis

**Figure 2:**
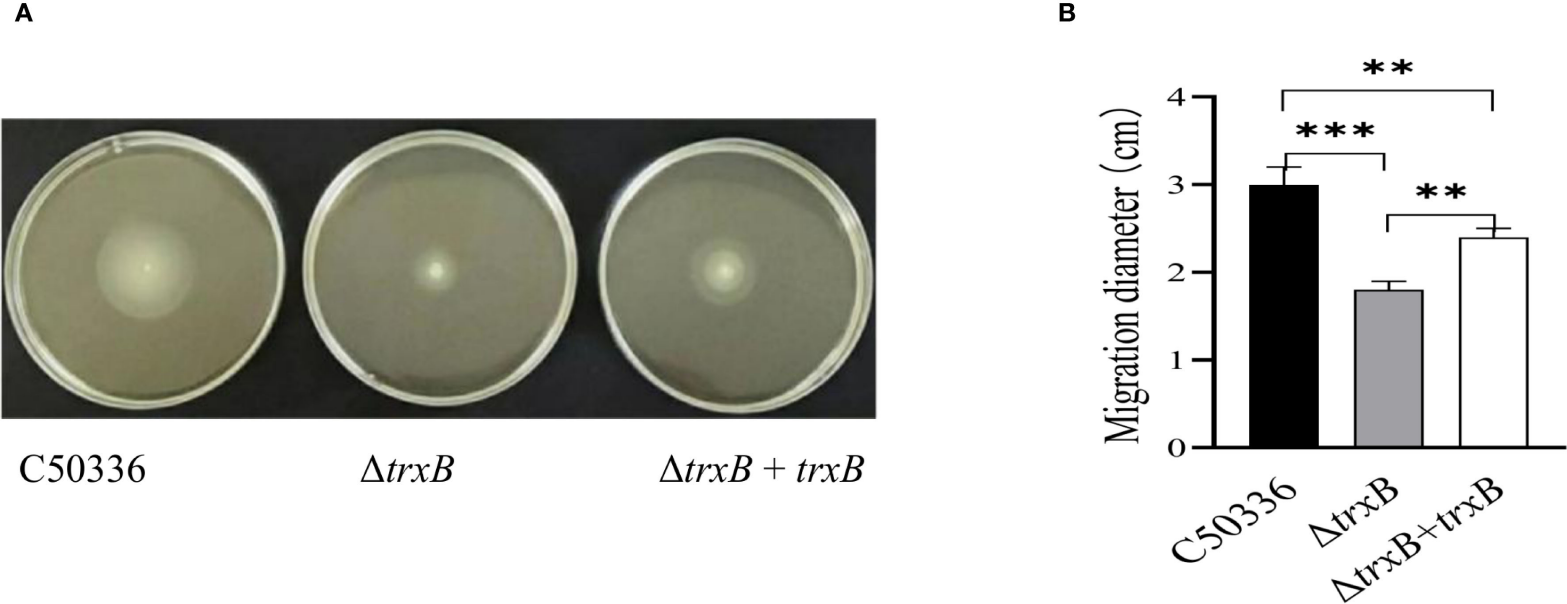
Motiity analysisi of each strain. **(A)** Five µL of the overnight culture of each strain was taken and inoculated by puncture at the center of the semi-solid LB plates containing 0.3% agar, and incubated at a constant temperature of 37 °C for 5 h The motility of each strain was analyzed by measuring the motility diameter of the strain on the medium plate after stabbing it into semi-solid medium. **(B)** Quantitative results of bacterial motility analysis. The mean of three replications of the experiment. (***p < 0.001).

The results of the motility skill assessment showed (**Figure 2A**) that the Δ*trxB* strain formed a much smaller swarming zone on semi-solid plates compared with C50336 and Δ*trxB*+*trxB* strains, and the motility of the strain after *trxB* gene restoration was restored compared to the *trxB* knockout strain, which was supported by the measurement of the diameters of the formed bacteria zones (**Figure 2B**). This result demonstrated that knockout of *trxB* could diminish the motility of *S*. Enteritidis.

### 3.3 The *trxB* gene deletion affects *S*. Enteritidis biofilm formation

**Figure 3:**
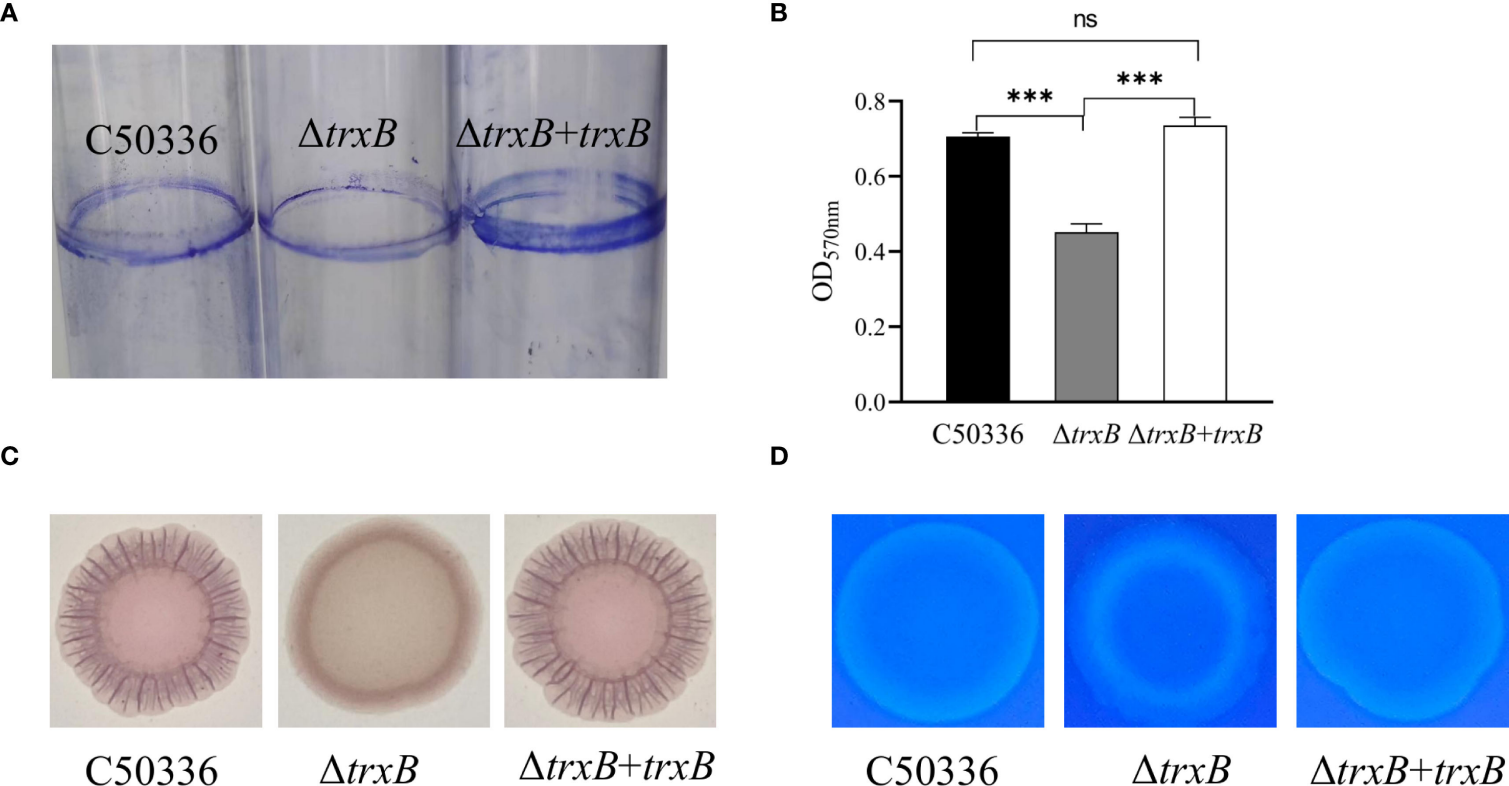
Biofilm formation capacity and composition analysis. **(A)** After three days of incubation of each strain in the medium, the biofilm attached to the test tubes was stained by 2% crystal violet solution to observe the formation of biofilm. **(B)** Qualitative detection of biofilm formation in 96- well microtiter plates, with absorbance measured at 570 nm (***p < 0.001). **(C)** Each strain was inoculated with Congo red (160 mg/L Congo red, 10 mg/L Caulmers Brilliant Blue) medium and incubated at 28 °C for 48 h The curli formation of the colonies of each strain were observed. **(D)** Each strain was inoculated into the medium containing fluorescent whitening agent (200 mg/L) and incubated at 28°C for 48 h, and the fluorescence intensity of the colonies with the fluorescent agent was observed under D366nm UV light. ns, not significant.

The biofilm formation ability of C50336, Δ*trxB*, and Δ*trxB*+*trxB* strains was examined, and the data showed (**Figure 3A**) that much less biofilm formation of Δ*trxB* strain was observed on tube surface compared to the C50336 and Δ*trxB*+*trxB* strains, while the biofilm formation ability of Δ*trxB*+*trxB* strains was restored compared to the *trxB* gene deletion strains via Crystal violet-staining. Quantitative results revealed that (**Figure 3B**) the *trxB* strain showed significantly lower absorbance values at OD_570nm_.

In order to clarify which components of the biofilm were affected by *trxB* knockout, all three strains were inoculated into Congo red medium and fluorescent-containing medium, respectively. As shown in **Figure 3C**, the Δ*trxB* strain formed smooth, pale pink colonies on Congo red medium, while the C50336 and Δ*trxB*+*trxB* strain showed much darker color and more ruffled colonies, indicating that the deletion of *trxB* reduced curli production. The fluorescence luminescence intensity of Δ*trxB* was significantly different compared to C50336, and the fluorescence intensity of the Δ*trxB*+*trxB* strain was restored compared with the Δ*trxB* strain (**Figure 3D**). All these results strongly indicate that the TrxB gene can influence the formation of *S*. Enteritidis biofilm.

### 3.4 TrxB plays an important role in the drug resistance of *S*. Enteritidis

The antibiotic sensitivity of the strains was assessed by MIC determination (**Table 3**). Compared with the wild-type strain, the *trxB* gene deletion strain showed an 8-fold increase in susceptibility to penicillin and mequindox, and a 4-fold increase to cefotaxime, cefuroxime, ampicillin, cotrimoxazole, norfloxacin, ciprofloxacin, enrofloxacin, oxfloxacin, lincomycin, and fosfomycin. These results strongly suggest that TrxB plays an important role in the drug resistance capacity of *S*. Enteritidis.

**Table 3:** MIC of *trxB* gene deletion strain.

Drug name	C50336	Δ*trxB*	Fold change
Cefotaxime Sodium	0.0625	0.015625	4
Cefuroxim	0.03125	0.0078125	4
Penicillin	8	1	8
Ampicillin	0.03125	0.0078125	4
Paediatric Compound Sulfamethoxazole Tablets	3200	800	4
Norfloxacin	0.03125	0.0078125	4
Ciprofloxacin	0.03125	0.0078125	4
Enrofloxacin	0.03125	0.0078125	4
Ofloxacin	0.0625	0.015625	4
Lincomycin	800	200	4
Mequindox	8	1	8
Fosfomycin	64	16	4

### 3.5 TrxB regulates the stress response of *S*. Enteritidis under various environmental conditions

**Figure 4:**
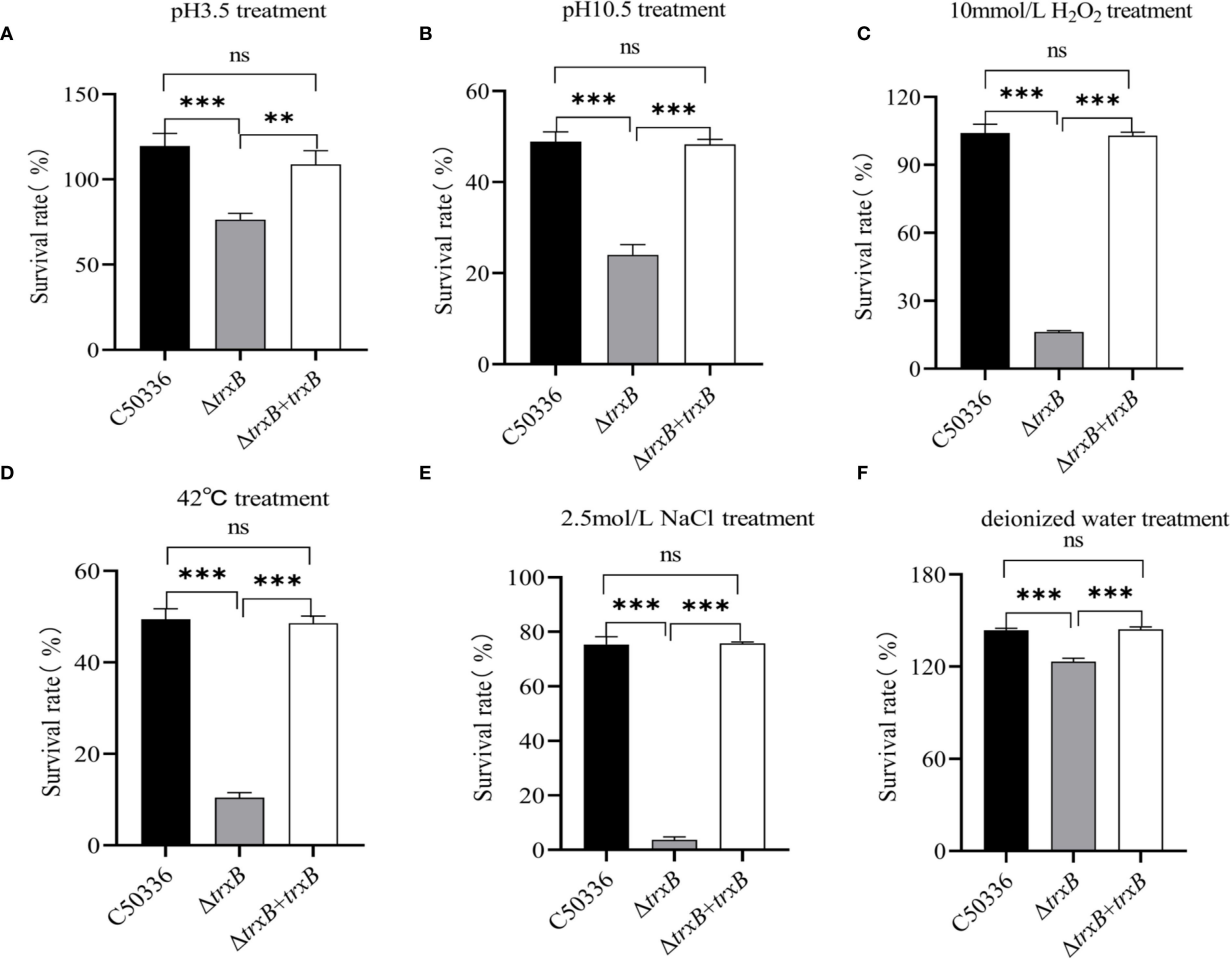
Bacterial stress-tolerance analysis. **(A)** The bacteria in logarithmic phase were transferred to acidic stress medium and the survival rate of each strain was calculated by bacterial counting after 1 hour of incubation. **(B)** The bacteria in logarithmic phase were transferred to alkaline stress medium and the survival rate of each strain was calculated by bacterial counting after 1 hour of incubation. **(C)** The bacteria in logarithmic phase were transferred to oxidative stress medium and the survival rate of each strain was calculated by bacterial counting after 10 min of incubation. **(D)** The bacteria in logarithmic phase were transferred to 42°C and the survival rate of each strain was calculated by bacterial counting after 1 hour of incubation. **(E)** The bacteria in logarithmic phase were transferred to hypertonic stress medium and the survival rate of each strain was calculated by bacterial counting after 1 hour of incubation. **(F)** The bacteria in logarithmic phase were transferred to hypotonic stress medium and the survival rate of each strain was calculated by bacterial counting after 1 hour of incubation. The data represents the average of 3 replicates (ns, not significant, **p < 0.01, ***p < 0.001).

The environmental tolerance results showed (**Figures 4A–F**) that the Δt*rxB* strain had a significantly lower survival rate in acidic, alkaline, oxidative, thermal, hypertonic, and hypotonic stress environments compared with the C50336 strain. Furthermore, the survival rate of Δ*trxB*+*trxB* strain in stressful environments was restored compared to Δ*trxB* strains. These results indicates that TrxB plays a critical role in regulating the stress response of *S*. Enteritidis.

### 3.6 The *trxB* gene deletion affects the adhesion, invasion and intracellular survival of *S*. Enteritidis

**Figure 5:**
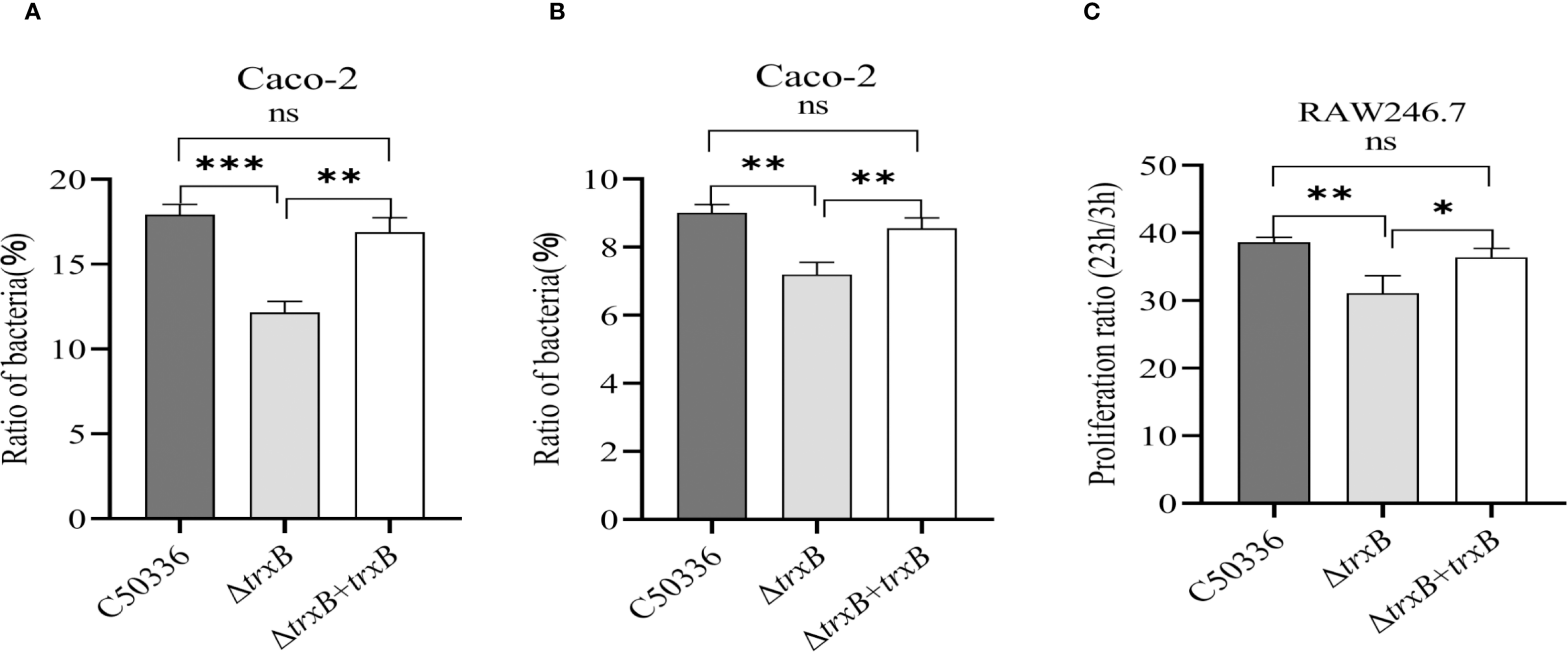
Adhesion, invasion and intracellular survival analysis of each strain. **(A)** Caco-2 cells were infected with Each strain and incubated for 1 hour at 37 °C Celsius in an incubator, followed by lysis of the cells using 1% Triton X-100, and the bacterial adhesion rate was calculated by drop plate counting. **(B)** The infection method for the invasion assay was the same as that for the adhesion assay. After 1 h of cell culture, the medium was replaced with DMEM medium containing gentamicin (100 μg/mL), and incubation was continued for 1 h 1 mL of 1% Triton X-100 was added to the lysed cells. Invasion of bacteria of each strain was calculated by drop plate counting. **(C)** The intracellular survival assay was performed in the same way as the invasion assay. The RAW264.7 cells were cultured in DMEM medium containing gentamicin (100 μg/mL) and lysed at 3 and 23 h after culture, respectively, and the intracellular viability of the bacteria was calculated by drop plate counting. The data represents the average of 3 replicates (*p < 0.05, **p < 0.01, ***p < 0.001).

Using Caco-2 and RAW264.7 cell models, we tested the adhesion, invasion and intracellular survival abilities of the Δ*trxB* mutant. The results showed (**Figures 5A, B**) that the adhesion and invasion rates of Δ*trxB* were significantly lower than those of C50336, and the survival of Δ*trxB* in cell-type macrophage of RAW264.7 lineage was significantly lower than that of C50336. The adhesion rate, invasion rate, and intracellular survival rate of the strain after *trxB* gene restoration were restored compared to the deletion strain. (**Figure 5C**). This indicates that TrxB affects the adhesion, invasion, and intracellular survival abilities of *S*. Enteritidis.

### 3.7 Deletion of the *trxB* gene attenuates the virulence of *S*. Enteritidis

**Figure 6:**
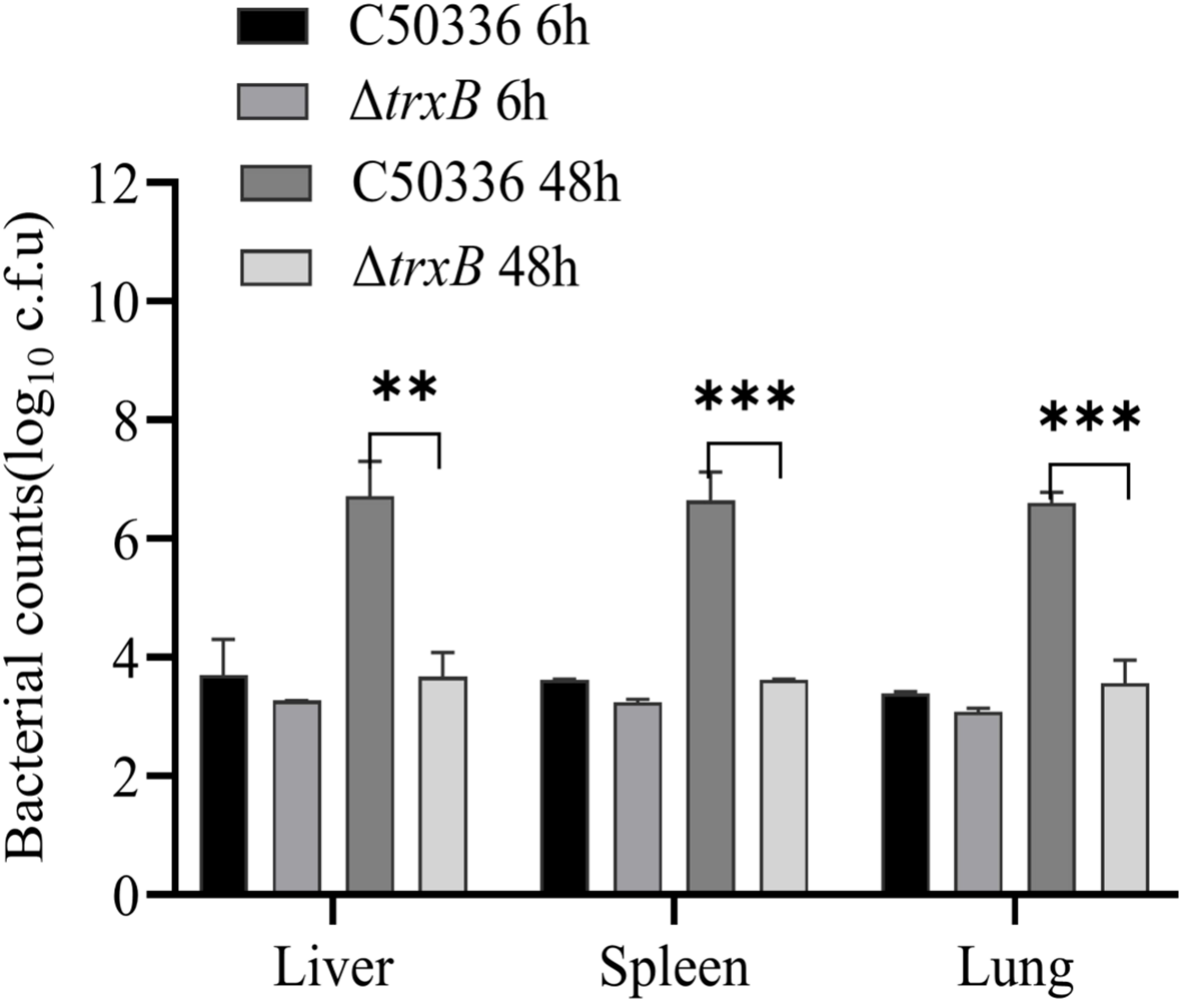
Distribution of bacteria in Kunming (KM) mice post infection. At 6 h and 48 h post-intraperitoneal injection of C50336 and Δ*trxB* into mice, the liver, spleen, and lungs were harvested and homogenized. The drop plate counting methods were used to calculate the bacterial load in tissues or organs. (**p < 0.01, ***p < 0.001).

The pathogenicity results of the Δ*trxB* strain results show (**Table 4**) that the LD_50_ value of the C50336 strain is 3.98 × 10^6^ CFU, while that of the Δ*trxB* strain is 5.13 × 10^8^ CFU, representing a 129-fold increase. The results of bacterial burden *in vivo* showed (**Figure 6**) that at 6 h post-infection, the bacterial loads in the liver, spleen and lungs from Δ*trxB* group were similar to those of C50336 group. However, at 48 h post-infection, the Δ*trxB* group showed significantly lower bacterial load in the liver, spleen and lungs compared to the C50336 strain. The above results suggest that deleting the *trxB* gene reduces virulence of *S*. Enteritidis.

**Table 4:** LD_50_ of C50336 and Δ*trxB* in KM mice.

Strain	Inoculation dose (CFU/mouse)	No. of deaths/total no. of mice	LD_50_ (CFU)
C50336	2.1×10^8^	5/5	3.98×10^6^
	2.1×10^7^	4/5	
	2.1×10^6^	4/5	
	2.1×10^5^	0/5	
	2.1×10^4^	0/5	
ΔtrxB	1.3×10^10^	5/5	5.13×10^8^
	1.3×10^9^	2/5	
	1.3×10^8^	0/5	
	1.3×10^7^	0/5	
	1.3×10^6^	0/5	

### 3.8 The *trxB* deletion leads to decreased expression of virulence genes

**Figure 7:**
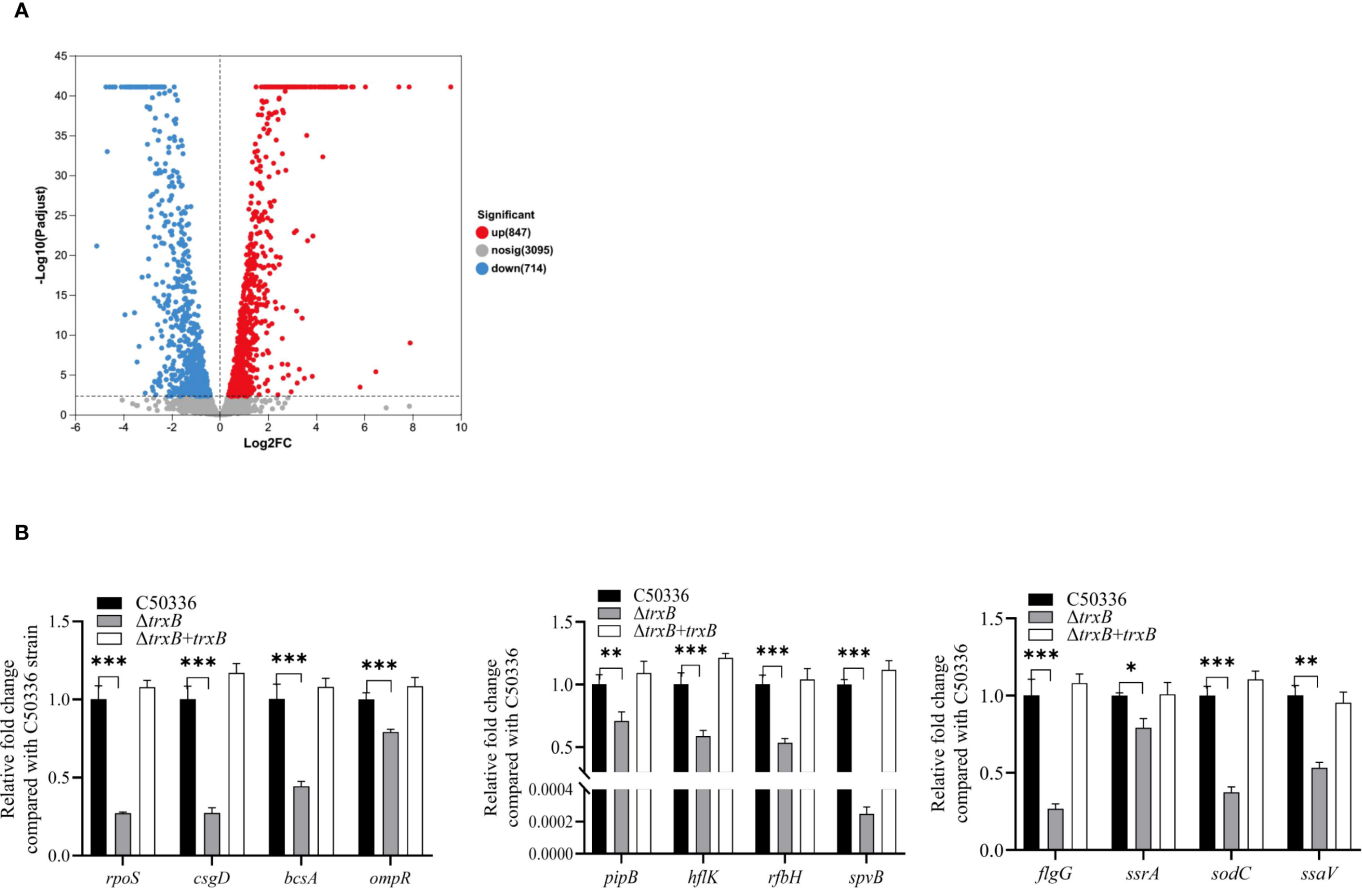
DEGs screening by RNA-seq and virulence gene expression analysis. **(A)** Screening for differential genes with expression changes in sequencing libraries. **(B)** The expression levels of virulence genes in C50336, Δ*trxB* and Δ*trxB* +*trxB* were detected by using qPCR, with 16S rRNA as the housekeeping gene. The data represents the average of 3 replicates (*p < 0.05, **p < 0.01, ***p < 0.001).

RNA-seq analysis identified 1561 differentially expressed genes (DEGs) in C50336 strain after *trxB* knockout, including 847 up-regulated and 714 down-regulated genes (**Figure 7A**).

Among these DEGs, we selected virulence-related genes and confirmed their expression levels by PCR. The results showed (**Figure 7B**) that the expression of *rpoS*, *csgD*, *bcsA*, *ompR*, *pipB*, *hflK*, *rfbH*, *spvB*, *flgG*, *ssrA*, *sodC* and s*saV* was significantly reduced. These results suggest that deletion of the *trxB* gene downregulates expression of multiple virulence genes in *S*. Enteritidis.

### 3.9 Deletion of the *trxB* gene provides good immune protection in mice

**Figure 8:**
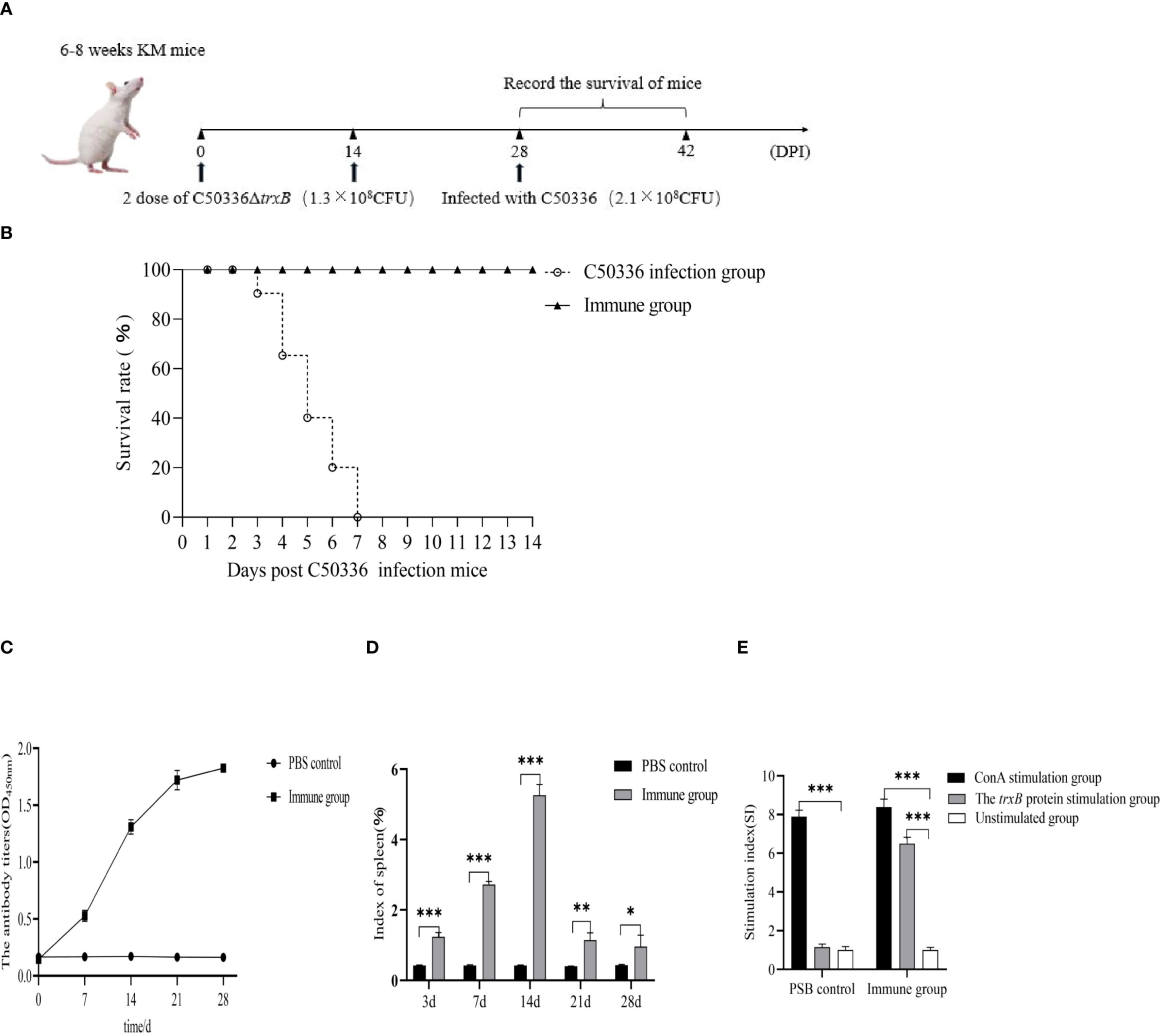
Determination of immunoprotective effect of the Δ*trxB* mutant. **(A)** Kunming mice were immunized with Δ*trxB* for 28 days and infected with C50336 (2.1×10^8^CFU) by intraperitoneal injection. Mice were observed daily for mortality. **(B)** Mouse survival curves. **(C)** KM mice were received orally with Δ*trxB*, using ELISA to determine the levels of IgG in mice at days 7, 14, 21 and 28 post-immunization. **(D)** Mice were weighed on days 7, 14, 21, and 28 post-immunization, and spleens were removed and weighed to calculate the spleen index. **(E)** Measurement of lymphocyte proliferation and transformation levels in the spleen of mice at 21 day post-immunization using the MTT assay, calculating the stimulation index (SI). (*p < 0.05, **p < 0.01, ***p < 0.001).

The mice were immunized with Δ*trxB* and challenged with C50336 on day 28 (**Figure 8A**). After infection, their clinical signs and survival were monitored. The immune group mice remained in usual behavior and all survived the challenge. In contrast, control mice showed dishevelled hair, reduced appetite, and lethargy by day 2 post-challenge, with deaths beginning on day 3 and reaching 100% mortality by day 7 (**Figure 8B**). According to the formula for calculating immune protection rate, the RPS for Δ*trxB* was 100%. This suggests that the Δ*trxB* provides effective immunoprotection against C50336 attacks.

Mouse blood serum was collected at different times after immunization for the detection of IgG antibody levels. The results showed (**Figure 8C**) that Δ*trxB* could induce antibody production on day 7 after immunization, and the antibody level was highest on day 14, indicating that *trxB* gene deletion induced humoral immune responses in mice, showing good immunogenicity.

Mouse spleens were collected at various time points after immunization to measure the splenic index. Results (**Figure 8D**) showed a significantly higher splenic index in immunized mice compared to controls, except at day 28 when no difference was observed. On day 21 post-immunization, splenocytes were collected for a lymphocyte proliferation assay. The data showed (**Figure 8E**) that the stimulation index (SI) index of the immunized group of mice was much higher than that of the unimmunized group.

## 4 Discussion

Thioredoxin reductase catalyzes the reduction of thioredoxin. It plays critical roles in thioredoxin-mediated redox homeostasis, cellular homeostasis, and oxidative stress tolerance (Lu and Holmgren, 2014b). It is well established that thioredoxin reductase has the ability to modulate cell growth and apoptosis by affecting gene transcription, playing a pivotal role in the differentiation and proliferation of mammalian cells (Kwon et al., 2003). Especially, it has gained wide attention in cancer research and has been proven to be a reliable target for anticancer drugs (Mohammadi et al., 2019). However, the literature onthioredoxin reductase in bacteria is rare. In this study, we manipulated the knockout of TrxB, the thioredoxin reductase gene, in *S*. Enteritidis, and found that this protein is able to cause multiple phenotype changes, including stress resistance and biofilm formation. Moreover, *trxB* deletion resulted in a significant reduction in the virulence of *S*. Enteritidis. More importantly, the *trxB* knockout strain, when used as an immunogen, provided promising immune protection for mice against wildtype *S*. Enteritidis infection.

The thioredoxin system regulates cell growth and death in mammals through redox regulation (Kwon et al., 2003). In cancer research, this protein has been reported to be closely related to apoptosis, and excellent therapeutic results have been achieved using its inhibitors in treating tumors in some studies (She et al., 2023). However, in this study, deletion of *trxB* did not significantly decrease bacterial growth, indicating that this protein does not affect the growth of *S*. Enteritidis.

Thioredoxin reductase can help organisms cope with oxidative stress by alleviating oxidative damage, which has been demonstrated in a variety of cancer cells and bacteria (Serata et al., 2012; He et al., 2023). In addition, the protein has been reported to be involved in other stress environments such as disulphide stress and acid stress (Uziel et al., 2004; Birk et al., 2012). In the present study, we found that knocking out *trxB* significantly decreased *S*. Enteritidis resistance to oxidative, acid, alkali, and heat stresses. This suggests that TrxB contributes to the bacterium’s tolerance to multiple environmental stresses.

Biofilm is an important accessory structure that helps bacteria survive harsh environments. It has also been reported to be closely associated with virulence (Karygianni et al., 2020). In a previous report, thioredoxin was reported to be a biofilm formation-associated factor in *Salmonella* using transposon insertion analysis (Chen and Wang, 2020). This finding is further supported by the data in this study, in which we found that the bacterial biofilm-forming capacity was significantly impaired after *trxB* deletion. Biofilms play a key role in bacterial resistance to antibiotics, so we also examined the drug resistance changes in bacteria before and after *trxB* deletion. We found that *trxB* deletion led to increased sensitivity of *S*. Enteritidis to multiple antibiotics, with up to 8-fold increase in sensitivity and the mechanism requires further investigation.

The oxidative stress tolerance is closely related to bacterial virulence, and as a key system regulating bacterial redox homeostasis, thioredoxin system’s involvement in bacterial virulence regulation has been demonstrated in *Edwardsiell*a, *Mycobacterium tuberculosis* (Olson et al., 2013), and *Acinetobacter baumannii* (May et al., 2019). Thioredoxin has also been reported to intervene in the intracellular infection and virulence of *Salmonella* typhimurium (Bjur et al., 2006). In this study, we observed that TrxB deficiency significantly decreased virulence in *S*. Enteritidis. This was evidenced by a more than 50-fold increase in LD_50_ in mice and an apparent reduction in bacterial load in the liver, spleen, and lung. These findings suggest that TrxB is an important virulence-associated factor of *S*. Enteritidis. Besides the role of antioxidant, thioredoxin functions as a cofactor for diverse enzymes and transcription factors, thereby exerting influence over their activity and function (Yang et al., 2024). We analyzed the transcriptomic changes before and after *trxB* deletion in *S*. Enteritidis, and found that up to 1561 genes were altered in expression, especially those associated with stress and virulence. Oxidative stress can activates transcription of multiple genes including *SPI* genes, and oxyR plays a key role in this process (Storz et al., 1990). In this study, we determined that TrxB knockout leads to down-regulation of 11 virulence gene, including *SPI* genes(*spvB*, *pipB*), biofilm related genes(*csgD*, *bcsA*, *ompR*). However, whether this downregulation effect is mediated by oxyR remains to be further investigated.

In *S*. Enteritidis, a wide range of virulence, metabolism and regulatory genes are employed for attenuated vaccine design (Siddique et al., 2024). In view of the marked reduced attenuation caused by *trxB* deletion in *S*. Enteritidis, we evaluated the potential of the *trxB*-deficient mutant as a vaccine. We found that immunization with this mutant could generate a promising immunity for mice against infection by wild type *S*. Enteritidis. Strong humoral immune responses and lymphocyte proliferation were also observed in immunized mice. These data suggest that TrxB is an potential target for *S*. Enteritidis design.

## 5 Conclusion

These studies indicate that TrxB is an important virulence factor of *S*. Enteritidis, and Δ*trxB* mutant exhibit strong immune protective effects in mice, making them potential targets for vaccine design.

## Data Availability

The datasets presented in this study can be found in online repositories. The names of the repository/repositories and accession number(s) can be found in the article/**Supplementary Material**.
