# Supplementary figures and images for: Characterization and immunoprotection of thioredoxin reductase TrxB knockout mutant of Salmonella Enteritidis
Source: Front Cell Infect Microbiol. 2025 Sep 17;15:1659729. doi: 10.3389/fcimb.2025.1659729 (PMC12484166; doi:10.3389/fcimb.2025.1659729)

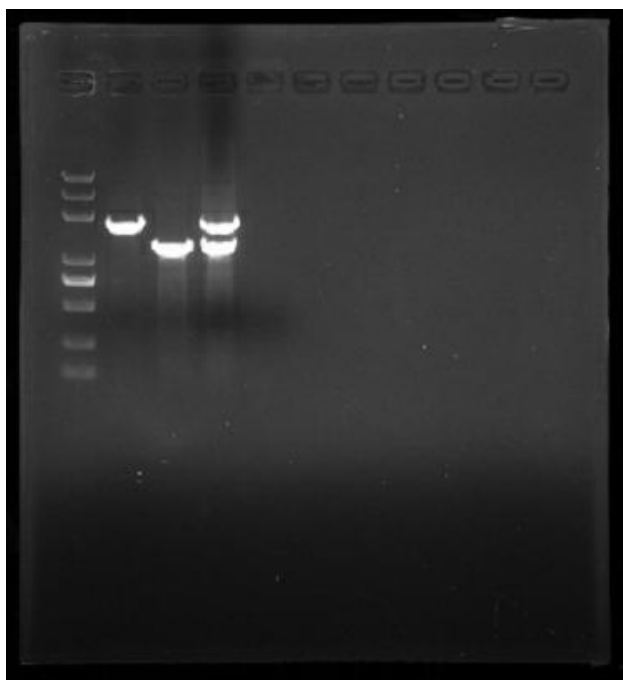

Supplement: Supplementary file 1 [file DataSheet1.pdf]

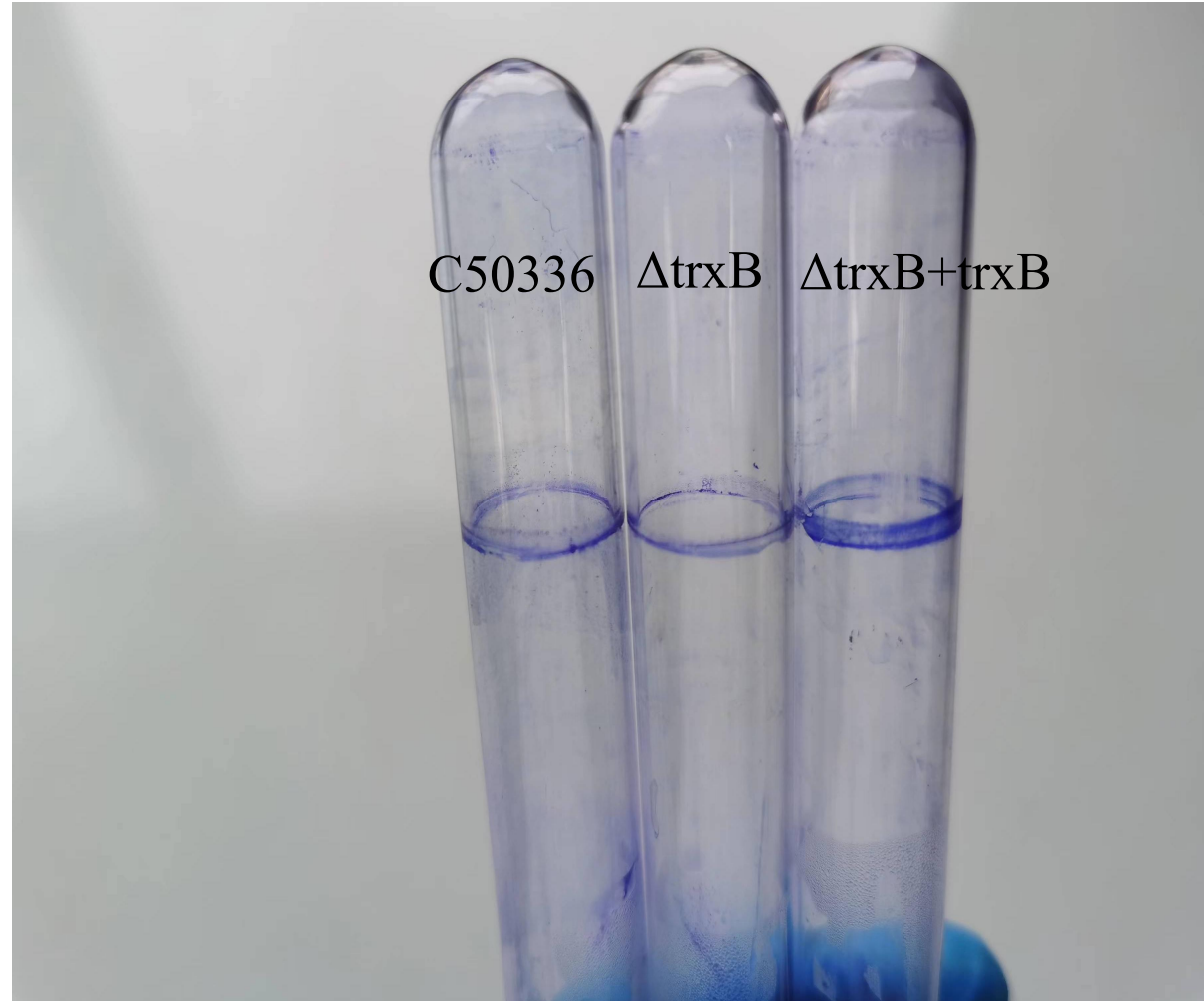

C50336

$\Delta\text{trxB}$

$\Delta\text{trxB}+\text{trxB}$

Supplement: Supplementary file 2 [file DataSheet2.pdf]

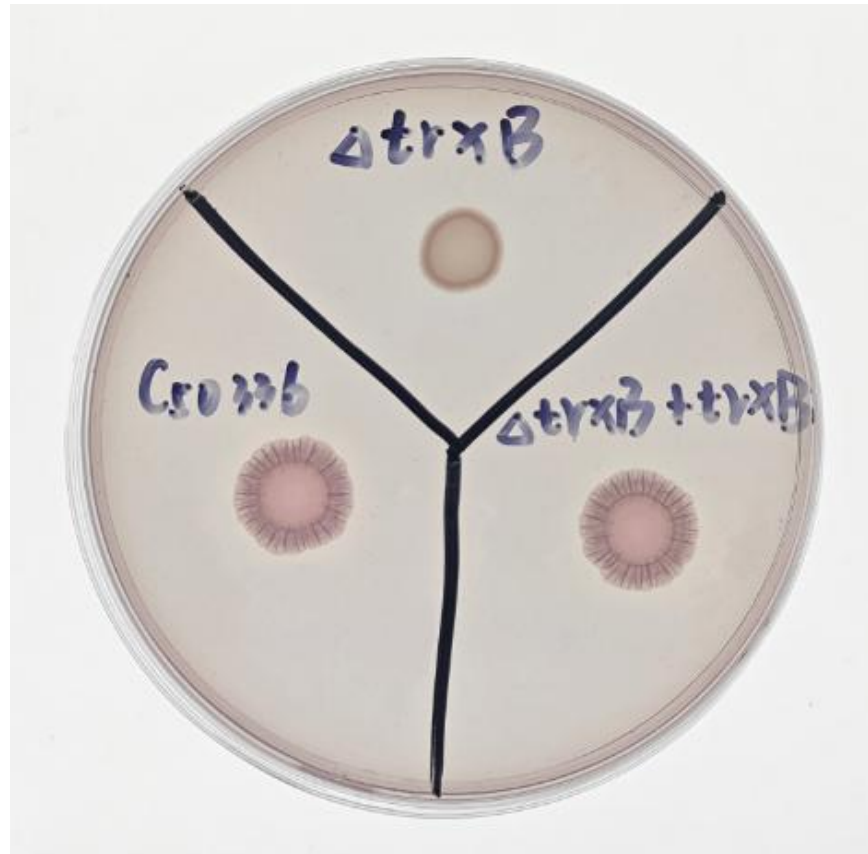

Supplement: Supplementary file 3 [file DataSheet3.pdf]

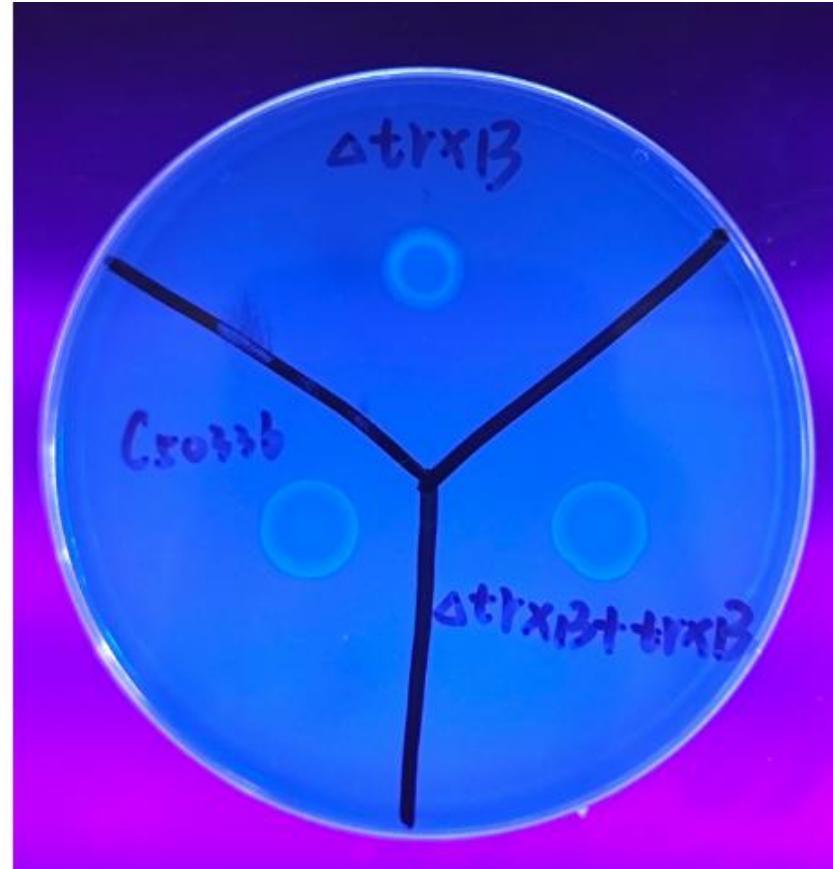

Supplement: Supplementary file 4 [file DataSheet4.pdf]
