# Supplementary material for: Characterization and immunoprotection of thioredoxin reductase TrxB knockout mutant of Salmonella Enteritidis
Source: Front Cell Infect Microbiol. 2025 Sep 17;15:1659729. doi: 10.3389/fcimb.2025.1659729 (PMC12484166; doi:10.3389/fcimb.2025.1659729)
Supplement: Supplementary file 5 [file DataSheet5.pdf]

KEGG enrichment analysis(C50336_vs_trxB_mRNA)

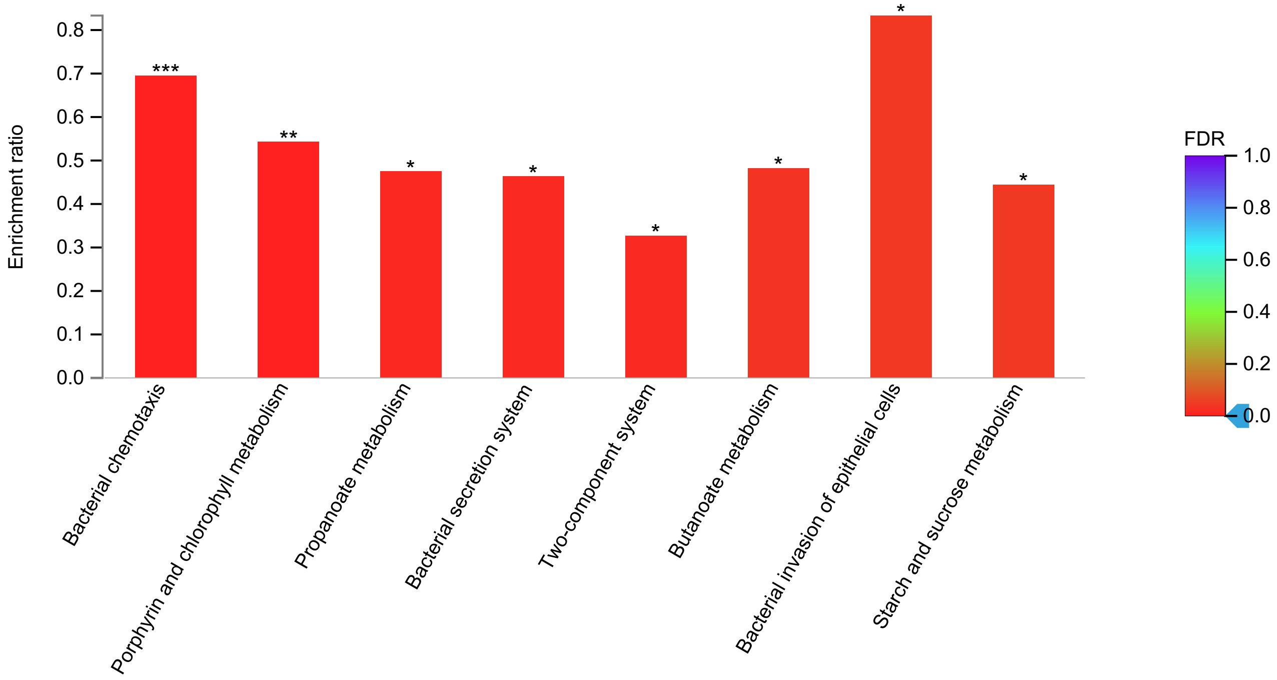

注：横坐标表示通路的名称；纵坐标表示富集率【基因集中注释到该pathway的基因数目（Sample number）与所有基因注释该pathway基因数目（Background number）的比值。Rich factor越大，表示富集的程度越大】。颜色表示富集的显著性即P-value，颜色越深表示该通路越显著富集，其中P-value<0.001的标记为***，P-value<0.01的标记为**,P-value<0.05的标记为*，右侧颜色梯度表示P-value大小。
